# On the Pb^2+^ Ions Adsorption onto *Prunus dulcis* Hull

**DOI:** 10.3390/molecules31132311

**Published:** 2026-07-01

**Authors:** Davide Lascari, Salvatore Giovanni Michele Raccuia, Paolo Lo Meo, Nicola Muratore, Salvatore Cataldo, Gabriele Lando, Marilena Tolazzi, Andrea Melchior, José Luis Barriada, Maria Martinez-Cabanas, Alberto Pettignano

**Affiliations:** 1 Dipartimento di Fisica e Chimica—Emilio Segrè, Università di Palermo, Viale Delle Scienze, I-90128 Palermo, Italy; davide.lascari@unipa.it (D.L.); nicola.muratore@unipa.it (N.M.); salvatore.cataldo@unipa.it (S.C.); 2 Dipartimento di Scienze Chimiche, Biologiche, Farmaceutiche ed Ambientali, Università Degli Studi di Messina, Viale F. Stagno d’Alcontres 31, I-98166 Messina, Italy; salvatoregiovannimichele.raccuia@unime.it (S.G.M.R.); gabriele.lando@unime.it (G.L.); 3 Dipartimento di Scienze e Tecnologie Biologiche, Chimiche e Farmaceutiche, Università di Palermo, Viale Delle Scienze, ed. 17, I-90128 Palermo, Italy; paolo.lomeo@unipa.it; 4 NBFC, National Biodiversity Future Center, Palermo, Piazza Marina 61, I-90133 Palermo, Italy; 5 Laboratorio di Tecnologie Chimiche, Dipartimento Politecnico di Ingegneria e Architettura, Università di Udine, I-33100 Udine, Italy; marilena.tolazzi@uniud.it (M.T.); andrea.melchior@uniud.it (A.M.); 6 Department of Chemistry, University of A Coruña, I-15071 A Coruña, Spain; jose.barriada@udc.es (J.L.B.); maria.martinez.cabanas@udc.es (M.M.-C.)

**Keywords:** *Prunus dulcis* hull, adsorption, remediation, lead ions, biomass, breakthrough curves

## Abstract

In this study, *Prunus dulcis* hull (PDH) has been used to develop a cost-effective and eco-friendly adsorbent material for the removal of Pb^2+^ ions from polluted waters. The PDH particles were characterized using various techniques, including ATR-FTIR spectroscopy, ion-selective electrode ISE-H^+^ potentiometric titrations, pH of point of zero charge (pH_pzc_) analysis, thermogravimetric analysis (TGA), and scanning electron microscopy with energy-dispersive X-ray spectroscopy (SEM-EDX). Single-batch adsorption experiments were conducted at different pH values, with pH 5.0 identified as the optimal initial pH in terms of Pb^2+^ adsorption performance of PDH. The study also evaluated the effects of temperature, ionic medium, and several organic ligands with different functional groups on the adsorption capacity of PDH. The results showed that PDH is an effective adsorbent for lead ions, with adsorption capacities (*q_m_*) ranging from 43 to 101 mg g^−1^ and an adsorption equilibrium time of approximately 750 min at room temperature. Additionally, column adsorption experiments demonstrated that PDH can be reused at least four times with minimal loss in performance. The adsorption behavior of PDH was comparable under both equilibrium (batch) and non-equilibrium (column) conditions, with the breakthrough time (BT_0.5_) values significantly affected by the background salts present in the toxic metal ion solution.

## 1. Introduction

Lignocellulosic feedstocks derived from agricultural and forestry activities represent a renewable and widely available resource with considerable potential across energy production, materials chemistry, and environmental remediation [1]. In the emerging bioeconomy, residual biomass is increasingly being exploited through co-firing with coal, dedicated biomass combustion systems, and advanced thermochemical conversion processes to generate heat, power, and liquid biofuels. Furthermore, biomass constituents, particularly cellulose and lignin, are valorised by integrated biorefineries that transform them into bioethanol, bioplastics, and other high-value fine and specialty chemicals [2,3,4,5]. Beyond their role in energy systems, these residues are precursors for functional materials such as biochar for soil amendment and wastewater treatment, activated carbon for water purification, and fiber-reinforced composites for lightweight structural applications [6,7,8].

The chemical versatility and renewable nature of lignocellulosic biomasses act as a promising solution for advancing sustainable chemical manufacturing and implementing circular economy principles [9]. Among their many applications, their use in environmental remediation has become strategic, especially in sustainable water treatment technologies. In response to growing global efforts to reduce water pollution and valorize agro-industrial by-products, increasing scientific interest has focused on lignocellulosic derivatives as cost-effective and environmentally friendly adsorbents for wastewater treatment [10]. Indeed, these materials naturally contain functional groups, including hydroxyl, carboxyl, and phenolic moieties, that provide strong affinity for a wide range of aqueous contaminants, including toxic metals, dyes, and organic pollutants [11,12,13].

Among water pollutants, toxic metal ions represent a particularly persistent and hazardous threat. Their non-biodegradable nature and tendency to bioaccumulate in food chains make them particularly problematic from both an ecological and public health perspective [14,15]. Pb^2+^ ion is considered one of the most dangerous toxic metal ions, as it can cause severe adverse effects even at trace concentrations. Chronic exposure to lead has been linked to neurological damage, cardiovascular disease, kidney dysfunction, and developmental disorders, especially in children [16,17]. For this reason, the development of ad hoc, scalable, and cost-effective purification technologies towards this toxic metal ion has become a key global priority, reflected in the goals outlined by the United Nations Sustainable Development Goals [18]. Indeed, conventional methods such as chemical precipitation, ion exchange, membrane filtration, and electrochemical treatment achieve regulatory targets [19] but, unfortunately, have several disadvantages like high operating costs, complex infrastructure, large volumes of secondary sludge, and declining performance at low contaminant levels [20,21].

Considering the pros and cons of available decontamination procedures, adsorption on agricultural and food waste biomasses would appear to be among the most cost-effective, as it operates under ambient conditions, requires no special reagents, generates a small amount of secondary waste, and allows for simple regeneration of the adsorbent [22].

Over the past decades, the study of the adsorption properties of different biomasses towards Pb^2+^ ions has been the subject of numerous articles and reviews published by researchers from all over the world [20,23,24,25] and our research group has given a contribute in this field by testing the adsorption properties of some of the most locally abundant biomasses, such as hazelnut (*Corylus avellana*) and almond (*Prunus dulcis*) shells [26], and *Opuntia ficus indica* cladodes [27].

In addition to the shells, the almond industry produces another secondary product, namely the hull. The hull is the outer covering of the kernel and the hard shell and represents the most abundant part of the fresh almond, accounting for about 50% of its weight. This percentage can be higher, up to 70%, depending on the almond variety [28].

Like other biomasses, the almond hull is mainly constituted by cellulose, hemicellulose, and lignin in different proportions that depend on different factors like cultivation conditions, harvesting methods, hulling procedures, almond variety, etc. [28,29]. Along with the expected water-insoluble macromolecular components, almond hull is rich in proteins, sugars, polyphenolic antioxidants, and even metal micronutrients [28,30,31,32], which suggests its possible use even as a functional ingredient for bread-making [31] and as an additive in livestock nutrition [28].

In 2023, the almond production in Italy was estimated at 77,680 tons [33]. It is also estimated that Italy produces ~1% of the world’s almonds [34], about 60% of which are produced in Sicily [35,36], making the almond-derived by-products highly available, in particular at the local level.

*Prunus dulcis* shells have been extensively investigated for adsorption applications in raw form, as chemically activated materials, and as biochar precursors [10,12,20,26,37,38]. Almond hull has received comparatively limited attention [23,39,40,41], and, to our knowledge, its adsorption properties towards Pb^2+^ ions have been investigated only by Nasseh et al. [23]. This article reports an in-depth study on the Pb^2+^ ion adsorption capacity of *Prunus dulcis* hulls (PDH) of *Romana* variety supplied by a local almond producer. The hull particles were prepared through a very simple process, without using chemicals. Therefore, they can be considered an economical and sustainable adsorbent, since they are an agricultural by-product and require low production costs.

This work aims not only to determine the adsorption capacity of PDH in water but also to understand how this material might behave, in terms of Pb^2+^ removal ability, in real aqueous matrices. For this reason, various scenarios were simulated by performing numerous kinetic and thermodynamic batch experiments in which were changed the initial pH (2 ≤ pH ≤ 6), the composition of solution (NaNO_3_ or NaCl 0.1 mol L^−1^, acetate, cysteine, or methylamine 5×10^−4^ mol L^−1^), the temperature (284.15 ≤ *T* (K) ≤ 303.15), and the Pb^2+^ ions concentration (30 ≤ *c*_Pb2+_ (mg L^−1^) ≤ 250). Furthermore, complementary column experiments were made to assess the recyclability of PDH particles over multiple adsorption–desorption cycles and to simulate a practical application of the adsorbent through continuous-flow fixed-bed columns, replacing the experimental conditions of batch tests. The PDH particles were extensively characterized (ATR-FTIR spectroscopy, SEM-EDX, TGA, and ISE-H^+^ potentiometry) to study their morphology, as well as the type, acid-base properties, and concentration of their functional groups and their involvement in the adsorption process of Pb^2+^ ions.

## 2. Materials and Methods

### 2.1. Reagents

Pb(NO_3_)_2_ (Sigma Aldrich, MA, USA, analytical grade) salt was used to prepare lead solutions. NaNO_3_ (Sigma Aldrich, MA, USA, 99.0%) and NaCl (Riedel-de Haën, Germany, 99.8%) salts were dried at 383.15 K for 2 h before use. Methylamine hydrochloride (MA, Alfa Aesar, MA, USA, 99%), sodium acetate (Ac, Carlo Erba, Italia, ≥99.5%), and L-cysteine (Cys, Fluka, Switzerland, ≥99.5%) were used as they were provided. HCl 0.975 N (Sigma Aldrich, MO, USA), HNO_3_ 1 M (Fluka Analytical, Switzerland), and NaOH 1 M (Fluka Analytical, Switzerland) were used to prepare diluted solutions, which were standardized against Na_2_CO_3_ (Merck, Germany, 99.9%) and C_8_H_5_KO_4_ (Merck, Germany 99.5%), respectively, and subsequently used to adjust the pH of Pb^2+^ solutions and in potentiometric titrations.

Standard solutions of Pb^2+^ (CertiPUR, Merck, Germany) and Ca^2+^ (Titrisol, Sigma Aldrich, MA, USA) 1000 mg L^−1^ in 2% HNO_3_ were used for instrument calibration. All the solutions of this work were prepared with freshly CO_2_-free ultrapure water (ρ ≥ 18 MΩ cm) and grade A glassware.

### 2.2. Adsorbent Preparation and Characterization

The PDH was collected in July 2023 in Sicily, near Palermo (Italy; 38°6′32.7″ N, 13°31′49.7″ E). They were cut into small pieces, dried in an oven at 333.15 K, carefully washed with tap water, and finally with ultrapure water. The washing procedure was stopped when TOC measurements of the supernatant confirmed the absence of organic matter. The PDH particles were dried at 333.15 K, ground with a domestic mill, and sieved with a sieve shaker Octagon Digital (Endecotts, UK). PDH particles with a size in the range 0.1< *x*/mm < 0.2 were collected for subsequent experiments.

The TGA of the PDH particles was performed by using a Thermogravimetric Analyzer Discovery, TGA 550 Discovery Series instrument (TA Instruments, DE, USA). In detail, 3–5 mg of PDH particles were previously dried at 323.15 K for 72 h and analyzed under an air flow of 60 mL min^−1^ with a balance purge of 40 mL min^−1^. The temperature range covered in the experiment was approximately 303.15–1173.15 K, with a heating rate of 10 °C min^−1^.

ATR-FTIR spectra of PDH particles (450 ≤ wavenumber (cm^−1^) ≤ 4000, spectral resolution = 16 cm^−1^, number of scans = 100), before and after the toxic metal ion adsorption, were recorded by using a PerkinElmer Spectrum Two instrument (Waltham, MA, USA). All the analyzed PDH samples were previously ground and dried at 383.15 K for 24 h.

PDH micrographs before and after the Pb^2+^ ions adsorption were acquired by a field-emission scanning electron microscope, JEOL model JSM-7610F Plus (JEOL Ltd., Tokyo, Japan) equipped with an energy dispersive X-ray spectroscopy probe (EDX, Oxford Instruments, UK) for semi-quantitative elemental analysis. A 15 kV voltage and a working distance of 15 mm were used, and the samples were previously coated with a 5 nm layer of gold to increase conductivity.

The acid–base behavior of the active sites of PDH surface was investigated through potentiometric titrations using a Metrohm 809 Titrando equipped with an Orion-ROSS combined “sure-flow” glass electrode (model 8172BNWP, uncertainty ± 0.15 mV) and an automatic burette (uncertainty ± 0.003 mL). The sure-flow electrode was selected to prevent junction clogging, a common issue when analyzing heterogeneous suspensions. The system was connected to a PC, with parameters such as titrant delivery, data acquisition, and e.m.f. stability, were monitored and controlled through the Metrohm TiAMO 2.5 software. Three potentiometric titrations were carried out under constant stirring on 25 mL aqueous suspensions containing 0.15, 0.20, and 0.30 g of PDH, hydrochloric acid (*c*_H_ = 0.01 mol L^−1^) to adjust the initial pH to approximately 2.0, and NaCl as background electrolyte to adjust ionic strength to a value of *I* = 0.10 mol L^−1^. The suspensions were constantly bubbled with saturated N_2 (g)_ (purity 99.99%) and titrated in thermostatted cells maintained at T = 298.15 ± 0.1 K with standard CO_2_-free sodium hydroxide solutions ([NaOH] = 0.1016 mol L^−1^) up to pH ~ 11.5.

Calibrations were performed at the same experimental conditions of the PDH suspensions to determine the standard electrode potential (*E*^0^), and the acidic junction coefficient (*j*_a_, *E_j_*_a_ = *j*_a_ [H^+^]).

The pH of the point zero charge (pH_pzc_) of PDH was estimated by the pH-drift method [42] in NaNO_3_ 0.1 mol L^−1^. Aliquots of 35 mL of solution at different pH levels between 2 and 10 were placed in different Erlenmeyer flasks with 25 mg of PDH particles. The suspensions were purged with purified N_2_ for 10 min, sealed, and shaken for 24 h. 

The drift method is one of the immersion techniques used in the literature, together with other methods like mass titrations, potentiometric mass titrations, and alkalimetric titrations, to determine the pH_pzc_. 

It is important to emphasize that the parameter thus calculated differs from the one originally proposed [43]. In particular, it provides the pH at which the material has an average surface charge equal to zero only under the experimental conditions (ionic medium, ionic strength) of the aqueous solutions in which it is determined (NaNO_3_ 0.1 mol L^−1^ in this work) [44].

### 2.3. Procedures for Batch and Column Adsorption Experiments

At first, batch adsorption tests of PDH particles towards Pb^2+^ ions were carried out at pH values of 2, 3, 5, and 6. To this end, ~20 mg of PDH particles were placed in different Erlenmeyer flasks containing 20 mL of Pb^2+^ ions solution 120 mg L^−1^, at *T* = 293.15 K. The suspensions were stirred at 180 rpm for 24 h with an orbital shaking incubator (Labbox instruments, Spain, mod. CTSI-070-001) and filtered through nylon syringe filters (SPHEROS, pore size = 0.45 μm) before measuring the Pb^2+^ ion concentrations in the collected supernatants.

The subsequent batch and column adsorption experiments were done at the initial pH = 5.

The kinetics of Pb^2+^ ions adsorption onto PDH particles were studied at pH = 5.0, *T* = 298.15 K, in NaNO_3_ and in NaCl 0.1 mol L^−1^. For each ionic medium, the experiment was performed twice by suspending ~19 mg of PDH particles in 50 mL of Pb^2+^ solution (*c*_Pb2+_ ≈ 24 mg L^−1^) in a thermostatted voltammetric cell under constant and regular stirring. The Pb^2+^ concentration in the suspension was measured at different contact times in the range 0–25 h through Differential Pulse Anodic Stripping Voltammetry (DP-ASV). The voltammetric apparatus, controlled by NOVA v. 1.10 software, was a Metrohm 663 VA stand combined with the Autolab potentiostat coupled with the IME663 interface. The VA stand was equipped with (i) a Multi-Mode Electrode Pro (Metrohm, Switzerland code 6.1246.120) working in the Static Mercury Drop Electrode (SMDE) mode, (ii) a glassy carbon auxiliary electrode (code 6.1247.000), and (iii) a double junction Ag/AgCl/KCl (3 mol L^−1^) reference electrode (code 6.0728.030). The experimental electrochemical conditions were the same reported in ref. [27].

Batch isotherm experiments were conducted in Erlenmeyer flasks by placing 20–200 mg of PDH particles in 20–50 mL of Pb^2+^ solution (*c*_Pb2+_ = 30–250 mg L^−1^) at pH = 5.0. The effects of ionic medium, organic ligands, and temperature were evaluated by using Pb^2+^ solution in pure water, in the ionic media NaNO_3_ and NaCl 0.1 mol L^−1^, in the presence of the organic ligands acetate (Ac), methylamine (MA), and L-cysteine (Cys) 0.5 mmol L^−1^, and in the temperature range 284.15–303.15 K. The suspensions were shaken for 24 h and filtered. After that, the supernatants were collected to measure the pH at adsorption equilibrium (pH_f_) and the Pb^2+^ ion concentration at equilibrium. A single batch of suspensions of the same background of the isotherm experiments and with the same amount of PDH particles was prepared and shaken for 24 h in order to measure the pH_f_ in the absence of Pb^2+^ ions.

The reusability and recyclability of the PDH were investigated by packing 19.9 mg of PDH particles into a glass column (2 cm diameter, 5 cm length) with glass beads at the top to prevent PDH particles movement. In the adsorption steps, 15 mL of lead solution 65 mg L^−1^, at pH = 5.0, was flowed at reflux (6 mL min^−1^ for 20 h). In the desorption steps, 15 mL of HCl 0.1 mol L^−1^ flowed at the same flow rate for 4 h. After each adsorption and desorption step, the adsorbent was rinsed with 100 mL of ultrapure water. Four adsorption–desorption cycles were performed. The fixed-bed column experiments were conducted in a down-flow configuration using a glass column (internal diameter: 1.5 cm; length: 18 cm) packed with 90 mg of PDH particles. The experimental setup was the same as that reported in ref. [27]. Breakthrough profiles for the adsorption of Pb^2+^ ions onto PDH were studied in ultrapure water, in NaNO_3_ 0.1 mol L^−1^, and in NaCl 0.1 mol L^−1^ aqueous solutions, at pH 5.0 and *T* = 293.15 K.

The Pb^2+^ ion concentration in the collected solutions was measured by Inductively Coupled Plasma Optical Emission Spectroscopy (ICP-OES) technique with a PerkinElmer, CT, USA, Model Optima 2100, equipped with an auto sampler model AS-90.

The pH of the Pb^2+^ solutions, before and after contact with the PDH particles, was measured with the same potentiometric apparatus previously described.

### 2.4. Kinetic, Isotherm, and Breakthrough Models

The kinetic experimental data (*t*, *q_t_*) were tentatively fitted with the most used models: the pseudo-first order (PFO) equation of Lagergren [45] (Equation (1)), the pseudo-second order (PSO) equation [46] (Equation (2)) and the Weber–Morris equation (Equation (3)) [47]:

(1) $$q_{t}=q_{e}\;(1-\;e^{-k_{1}t})$$

(2) $$q_{t}=\frac{q_{e}^{2}\;k_{2}\;t}{1+q_{e}\;k_{2}t}$$

(3) $$q_{t}=k_{i}t^{0.5}$$

where *q_t_* and *q_e_* represent, respectively, the adsorption capacity of PDH particles (mg g^−1^) at time *t* and at the equilibrium; *k*_1_ (min^−1^) and k_2_ (g mg^−1^ min^−1^) are the rate constants of adsorption., *k_i_* (mg g^−1^ min ^−0.5^) is the intraparticle diffusion constant.

The isotherm experimental data were fitted to the Freundlich [48] (Equation (4)) and Langmuir [49] (Equation (5)) models:

(4) $$q_{e}=\;K_{F}\;c_{e}^{1/n}$$

(5) $$q_{e}=\frac{q_{m}\;K_{L}\;c_{e}}{1+K_{L}\;c_{e}}$$

where *q_m_* (mg g^−1^) is the maximum adsorption capacity of the PDH particles, *c_e_* (mg L^−1^) is the Pb^2+^ concentration at equilibrium, *K_F_* (L^1/n^ g^−1^ mg^1−1/*n*^) and *K_L_* (L·mg^−1^) are the constants of the Freundlich and Langmuir models, respectively.

The amount of Pb^2+^ ions adsorbed at different contact times *t* (*q_t_*) was calculated by Equation (6):

(6) $$q_{t}=\;\frac{V\;(c_{0}-c_{t})}{m}$$

where *V* (L) is the volume of the Pb^2+^ ion solution, and *m* is the mass of PDH particles (g); *c*_0_ and *c_t_* are the Pb^2+^ ion concentrations in the solutions (mg L^−1^) at *t* = 0 and *t* = *t*, respectively. The same equation was used to calculate *q_e_*, replacing *c_t_* with the equilibrium concentration (*c_e_*).

The Langmuir constant values (*K_L_*/L mol^−1^) [50] at different temperatures were used to calculate the thermodynamic parameters Δ*G*^0^ (kJ mol^−1^), Δ*H*^0^ (kJ mol^−1^), and Δ*S*^0^ (kJ mol^−1^ K^−1^) by using Gibbs (Equation (7)) and van’t Hoff (Equation (8)) equations. The following assumptions were made: (i) the adsorption is reversible, (ii) the adsorption stoichiometry does not change, (iii) the adsorption equilibrium was reached during the experiments [50].

(7) $${∆G}^{0}=-RT\ln K_{L}$$

(8) $$\ln K_{L}=-\frac{{∆H}^{0}}{RT}+\frac{{∆S}^{0}}{R}$$

where *R* is the universal gas constant 8.314 J mol^−1^ K^−1^ and *T* is the temperature in K.

The nonlinear forms of the logistic (Equation (9)), Gompertz (Equation (10)), and Log-Gompertz (Equation (11)) models [51] were used to fit the breakthrough curves:

(9) $$\frac{c_{t}}{c^{0}}=\frac{1}{1+exp(a-bt)}$$

(10) $$\frac{c_{t}}{c^{0}}=exp\left[-exp(\alpha_{G}-\beta_{G}t)\right]$$

(11) $$\frac{c_{t}}{c^{0}}=exp\left[-exp(\alpha_{LG}-\beta_{LG}lnt)\right]$$

where *a* and *b* (min^−1^) represent the parameters of the logistic model, whereas $\alpha_{G}$, $\beta_{G}$ (min^−1^) and $\alpha_{LG}$, $\beta_{LG}\;$((ln min)^−1^) are the respective parameters of the Gompertz and Log-Gompertz models. Equation 9 is equivalent to the simplified Bohart–Adams model [52], which is also mathematically analogous to the Thomas and Yoon–Nelson [51]. Accordingly, the parameters *a* and *b* can be correlated to those used in the Thomas and Yoon–Nelson formulations, as previously documented [51]. While the logistic-based models (i.e., simplified Bohart–Adams, Thomas, and Yoon–Nelson) share a common derivation and reduce to an identical sigmoidal function, the Gompertz and Log-Gompertz models are empirically derived and proposed for fitting adsorption data without an explicit mechanistic basis [53].

Fitting of experimental data to kinetic, isotherm, and breakthrough models was performed using the OriginLab suite 2025 software (OriginLab Corporation, Northampton, MA, USA).

## 3. Results and Discussion

### 3.1. Characterization of PDH Particles

**Figure 1:**
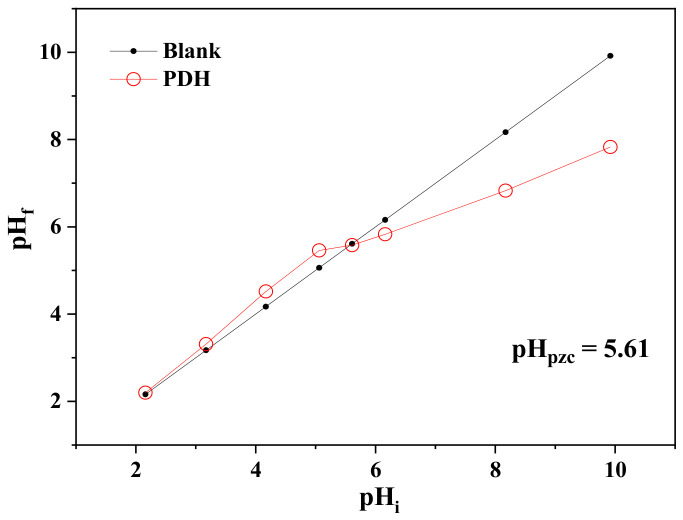
pH_pzc_ of PDH particles in NaNO_3_ 0.1 mol L^−1^.

The pH_pzc_ of PDH particles measured in NaNO_3_ 0.1 mol L^−1^ was 5.61 (see Figure 1), indicating a net positive or negative charge on the particle’s surface at pH values below or above this value, respectively. According to IUPAC recommendations [43], a rigorous determination of the point of zero charge requires a direct surface-charge characterization and assessment of ionic-strength effects. Therefore, it should be emphasized that the here reported pH_pzc_ value can be used only as a comparative descriptor of the acid–base behavior of the adsorbent under fixed ionic-medium conditions.

The net surface charge depends on the protonation/deprotonation equilibria of the functional groups of the numerous macromolecules present in the biomass. The acid-base equilibria of biomass, which represent a challenging task, were studied through a series of ISE-H^+^ potentiometric titrations carried out on PDH particle suspensions in NaCl at the same ionic strength of pH_pzc_ determination.

The analysis of the potentiometric titrations was carried out using the BSTAC software (version 4.0) [54]. During the data elaboration, several models were tested, considering different pH ranges and assuming the presence of a different number (from 1 to 4) of monoprotic functional groups. For each model, the acidic dissociation constant(s) and the site concentration (*c*_PDHi_, mmol L^−1^) for each titration were determined. The material site density (PDH_i_, mmol g^−1^) was estimated, for each titration, by using Equation (12):

Site density (PDH_i_, mmol g^−1^) = *c*_PDHi_ (mmol L^−1^) × Volume (L)/PDH mass (g) (12)

This calculation was used as a measure of the reliability of the data analysis.

As a whole, four different models were obtained considering one, two, three, and four monoprotic functional sites. Among them, the most reliable results were obtained elaborating data within the 2 ≤ pH ≤ 5 range. The choice of using such a pH range also depends on the fact that the recovery of metal cations is achieved in acidic conditions (pH < 6).

The dissociation constants and the site concentrations (*c*_PDH1_) are summarized in Table 1. Considering the acidic constant value obtained, log K^H^ = 3.73 ± 0.02 (95% C.I.), these functional groups likely refer to carboxylate moieties. The material site density obtained by considering all the titrations is PDH_1_ = 0.14 ± 0.01 mmol g^−1^ (95% C.I.).

As far as other models are concerned, the obtained results are listed in Table S1 of the Supplementary Materials and show that site concentrations estimated for one of such sites (i.e., PDH_4_) are too high to be reliable, as values obtained were around 50–150 mmol L^−1^. Considering that the PDH_4_ site acidic constant is ~ 12, namely, undergoing deprotonation in very alkaline conditions, it was confirmed that sorbent particles partially dissolved in these extreme pH conditions, as reported in refs [55,56,57] for lignocellulosic materials.

**Figure 2:**
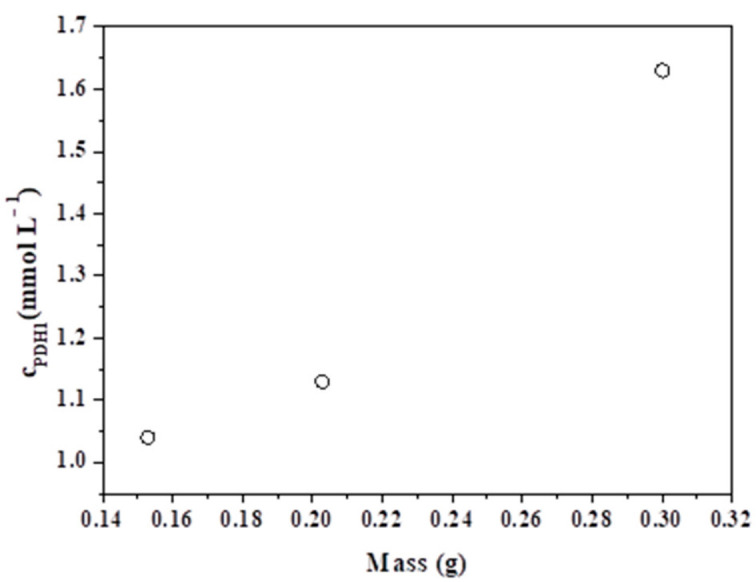
Scatter plot of *c*_PDHi_ (mmol L^−1^) vs. PDH mass (g) assuming only one monoprotic functional group (PDH_1_) in NaCl 0.1 mol L^−1^, at *T* = 298.15 K, pH range: 2–5.

To provide a chemically meaningful modeling, the material site density should remain constant regardless of the amount of biomass used within experimental error. Therefore, *c*_PDH1_ is expected to increase proportionally with mass, resulting in data points aligned along a linear trend. The scatter plot (Figure 2) reporting the site concentration (*c*_PDH1_, mmol L^−1^) obtained as a function of PDH mass used for the titrations (0.15289 g, 0.20279 g, and 0.30013 g) shows a clear correlation between the two variables, thus confirming the robustness of the one-site model.

**Figure 3:**
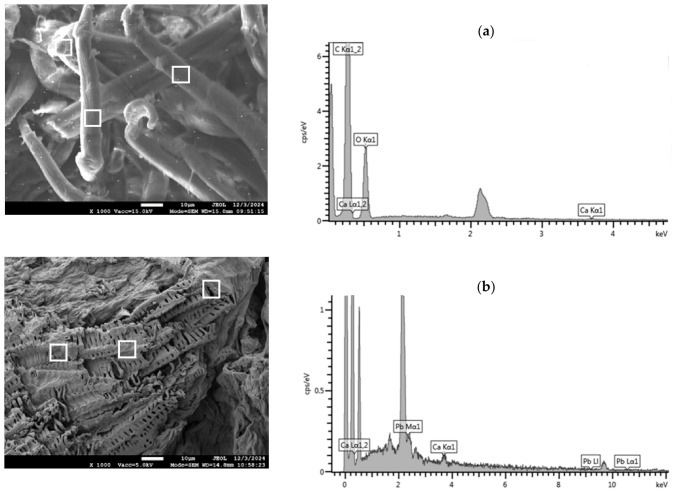
SEM micrographs obtained at 1000× magnification (the white squares indicate the acquisition points of the EDX spectra) and EDX spectra of PDH particles as they are (**a**) and after Pb^2+^ ions adsorption (**b**).

SEM micrographs at 1000× magnification and EDX spectra of PDH before and after Pb^2+^ ion adsorption are shown in Figure 3. The micrographs showed a biomass consisting of particles with irregular and thread-like shapes and a surface that becomes more wrinkled and corrugated after the Pb^2+^ ions adsorption. The rough elemental composition of PDH in % (*w*/*w*), as an average of three EDX spectra (see Table 2), confirmed the toxic metal ion adsorption.

**Figure 4:**
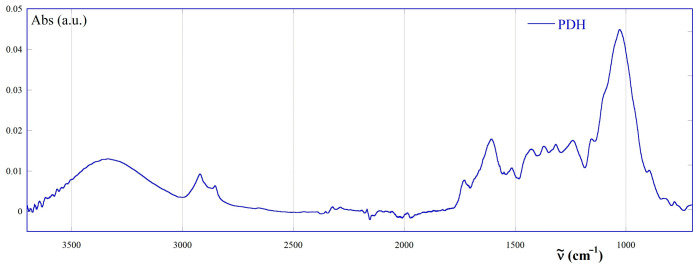
ATR-FTIR spectrum (3700~700 cm^−1^) of the PDH biomass.

The ATR-FTIR spectrum of the PDH (Figure 4) is perfectly consistent with the one already published by Salgado-Ramos et al. [32] and by Scappaticci et al. [58]. The main signals present in the spectrum are summarized in Table 3. Tentative attributions can be made on the grounds of literature reports; however, caution is needed in accomplishing the task due to the complex nature of the biomass. For instance, close inspection of the peak centered at 1606 cm^−1^, attributed to aromatic C=C str., appears indeed to be the convolution of several bands centered at higher wavenumbers, which can be reasonably attributed to the protein content (namely, Amide-I-like bands). In a similar way, the complex band system in the 1500~1100 cm^−1^ region derives from the superimposition of the fingerprint vibrational modes of lignin, carbohydrates, and polyphenol constituents, mainly associated with the aromatic C-C and the C-O stretching vibrations. Conversely, the strong signal centered at 1028 cm^−1^ can be confidently attributed to the macromolecular polysaccharide backbone (cellulose, hemicellulose).

**Figure 5:**
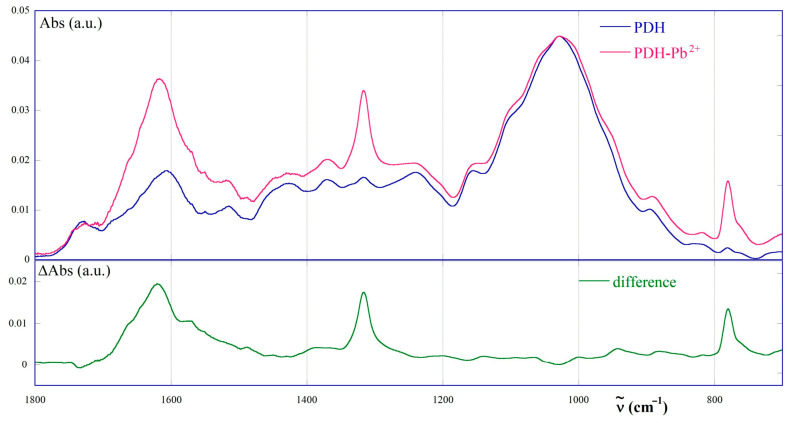
ATR-FTIR spectra (1800~700 cm^−1^) of PDH before (blue) and after (red) the adsorption of Pb^2+^ ions, and relevant difference spectrum (green) after suitable baseline correction and normalization with respect to the cellulose peak (1028 cm^−1^).

Interaction of the biomass with the Pb^2+^ ion causes subtle but interesting modifications in the FT-IR spectra (Figure 5). In fact, along with a strong enhancement, a slight blue-shift in the aromatic str. band (from 1606 to 1617 cm^−1^), two new signals appear, centered at 1315 and 780 cm^−1^. These findings tentatively suggest that the metal ion might preferentially interact with the aromatic moieties of lignin and other insoluble polyphenols present in the biomass, according to the model proposed by Soria et al. [59] for the interaction of metal cations with biochars. This might involve the occurrence of both π-cation and OH-cation interactions. In fact, the aforementioned signals at 1315 and 780 cm^−1^ are consistent with the aromatic C-O vibrational modes and the aromatic C-H out-of-plane bend, respectively [60,61,62]. Nevertheless, further interactions cannot be ruled out.

**Figure 6:**
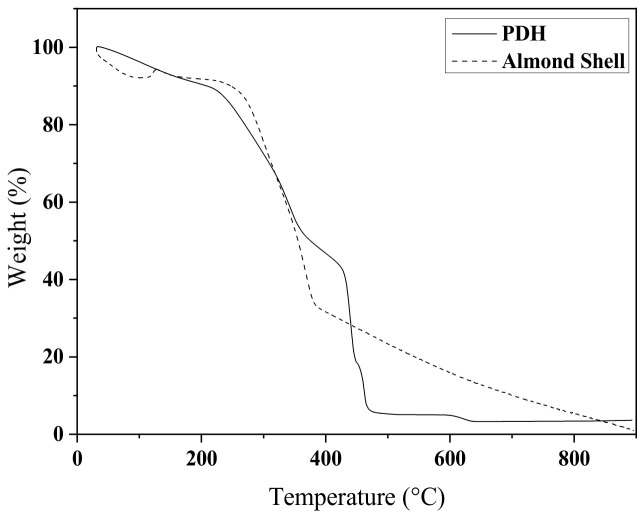
TGA curves of PDH (straight lines) and of the almond shell [26] (dashed lines) particles.

Further information on the PDH composition was obtained from its thermal decomposition profile in an oxidizing atmosphere. The TGA curve of PDH particles in air is reported in Figure 6. For comparison purposes, in the same graph is shown the TGA curve of shell particles of the same almond cultivar previously investigated [26].

The TGA curve of PDH particles showed an initial mass loss at low temperatures (T < 230 °C), attributable to the removal of moisture and water trapped in the organic matrix, in line with observations for other lignocellulosic biomasses under oxidizing conditions [63]. A second degradation stage between approximately 230 °C and 370 °C was attributed to the decomposition of the most thermolabile organic compounds, primarily hemicellulose, cellulose, and volatile components, which are typical of lignocellulosic biomasses heated in air [64]. At higher temperatures, a third decomposition step was observed up to about 430 °C, mainly associated with cellulose and, to a lesser extent, lignin degradation. Beyond this temperature, the material undergoes progressive carbonization, which ends around 470 °C, like in other lignocellulosic biomasses with comparable lignin and cellulose contents [63]. In the range of 550–650 °C, a slight additional mass loss can be observed, suggesting secondary degradation of the residual carbon or oxidation of more stable organic species. Above this temperature, the PDH sample maintains a nearly constant mass, corresponding to about 3.4% of the initial weight, indicating the complete decomposition of the organic components.

A comparison between the TGA curves of PDH and almond shell particles under the same experimental conditions highlights marked differences in their thermal behavior, directly related to their distinct chemical composition and lignocellulosic structure. In particular, PDH shows a more concentrated degradation within the low and intermediate temperature range (230–430 °C), with faster mass loss and a smaller amount of solid residue. This degradation profile reflects a higher content of labile polysaccharidic fractions, mainly hemicellulose and cellulose, and a lower lignin content.

The more pronounced decomposition in the second stage and the lower carbonaceous residue (~3%) are indicative of a structure richer in oxygenated components and less stabilized, consistent with the more fibrous and less lignified nature of PDH particles [38,65].

Conversely, the almond shell exhibits a more gradual and extended degradation profile, with a third stage reaching much higher temperatures (≈900 °C). These features indicate a relatively higher lignin content, a component known for its greater thermal resistance and slower oxidative behavior [63,64]. Lignin, indeed, degrades progressively over a wide temperature range, producing a more stable char that oxidizes only above 600 °C [38,63,64,65], confirming that the relative proportion of cellulose, hemicellulose, and lignin is the key factor governing the oxidative thermal behavior of biomasses.

### 3.2. Data Analysis of the Adsorption Kinetics

**Figure 7:**
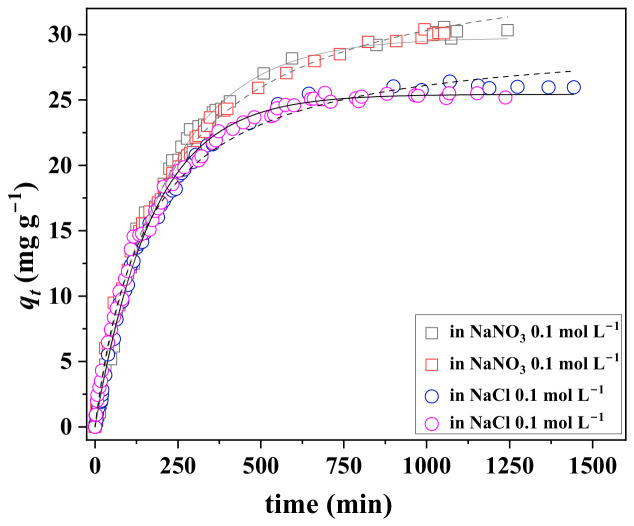
Dependence of *q_t_* (mg Pb^2+^ adsorbed/g of adsorbent) on contact time of PDH particles with aqueous NaNO_3_ (☐) and NaCl (◯) solutions at *I* = 0.1 mol L^−1^, at pH = 5 and *T* = 298.15 K. Data are fitted with PFO (continuous line), PSO (dashed line) kinetic models.

The adsorption kinetics of Pb^2+^ ions onto PDH particles have been studied at pH = 5.0, in NaNO_3_ and in NaCl 0.1 mol L^−1^, and at *T* = 298.15 K. All the experiments were carried out in a voltammetric cell at the same Pb^2+^ concentration and adsorbent–adsorbate ratio (see Section 2.3). The experimental data were processed with the most used PFO and PSO kinetic equations, whose refined parameter values are reported in Table 4 together with the statistical parameters adj R^2^ and std. dev. of fits. Both models fit the collected data very well with no significant differences in goodness of fit (see Figure 7). However, the PFO model was preferred based on the refined *q_e_* values, which were much closer to the *q_e_* _exp_ in both the ionic media investigated.

The adsorption equilibrium was reached after about 750 min with a slightly faster adsorption process in NaCl (*k*_1_ = 4.69×10^−3^ and 5.82×10^−3^ min^−1^ in NaNO_3_ and NaCl 0.1 mol L^−1^, respectively). The largest difference between the ionic media was found in terms of adsorption ability at equilibrium, which was reduced by ~5 mg g^−1^ by the addition of NaCl. It is likely attributable to the different chemical speciation of lead in the two ionic media. In particular, in NaNO_3_ 0.1 mol L^−1^, almost the 100% of the toxic metal ion is present as aquo ion. In NaCl 0.1 mol L^−1^, at the experimental conditions of the kinetic experiment, the percentage of aquo ion reduces to ~50% due to the formation of Pb–Cl species (PbCl^+^, PbCl_2_, PbCl_3_^−^) [66]. The different sizes and charges of these species result in a reduced affinity for the PDH binding sites, leading to lower *q_e_* values.

**Figure 8:**
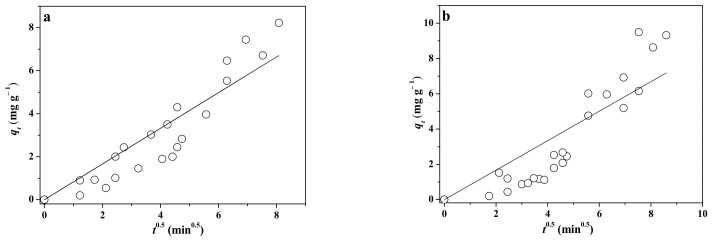
Weber–Morris plots for the kinetic adsorption of Pb^2+^ ions onto PHD particles from solution containing NaCl 0.1 mol L^−1^ (**a**) and NaNO_3_ 0.1 mol L^−1^ (**b**), at pH = 5.0, and at *T* = 298.15 K.

To elucidate whether intraparticle diffusion acted as the rate-limiting step, the kinetic data were also analyzed using the Weber–Morris model in its original formulation [47,67] (see Figure 8). Theoretically, if the process is strictly controlled by diffusion, a linear relationship between *q_t_* and t^0.5^ must be observed in the initial stage of adsorption (fractional uptake *q_t_*/*q_e_* < 0.3).

Among the two investigated systems, better linearity in the Weber–Morris plot (adj. R^2^ = 0.8438) was observed only for the kinetic data collected in NaCl. However, the lower linearity obtained in NaNO_3_ (adj. R^2^ = 0.7536) does not exclude the occurrence of diffusion-related phenomena but suggests that additional interaction mechanisms like ionic exchange or physical adsorption contribute to the adsorption behavior. These mechanisms remain consistent with a predominantly physisorption-driven process (see Section 3.4).

### 3.3. Data Analysis of the Adsorption Equilibria

**Figure 9:**
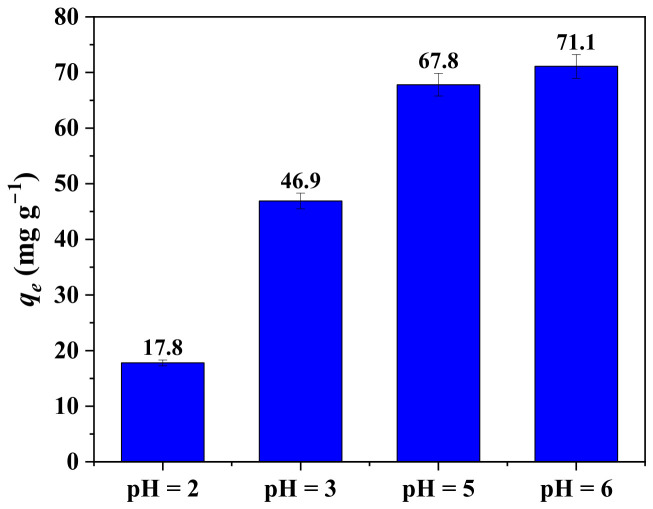
*q_e_* values of Pb^2+^ ions adsorption onto PDH particles in the pH range 2.0–6.0. Experimental details: ~20 mg of PDH particles in 20 mL of Pb^2+^ 120 mg L^−1^, at *T* = 293.15 K.

Some single batch adsorption tests were carried out with Pb^2+^ ion solutions at pH = 2.0, 3.0, 5.0, and 6.0 and at *T* = 293.15 K using the same adsorbent/adsorbate ratio at which the adsorbent was saturated (*q_e_* → *q_m_*). The *q_e_* value gradually increases with the increasing of pH and reaches a quite stable value starting from pH = 5.0 (see histogram of Figure 9). The experiments were carried out up to pH = 6 to prevent the formation of low-solubility hydrolytic species of Pb^2+^ ions. The most plausible reasons for this trend are the competition between H^+^ ions and Pb^2+^ ions for PDH binding sites at low pH values and the gradual deprotonation of PDH binding groups with increasing pH.

pH = 5 was chosen as the initial pH for all isotherm experiments. Furthermore, the pH of all suspensions at adsorption equilibrium was measured to obtain further information about the adsorption mechanism and to confirm the absence of low-soluble hydrolytic species of lead that would distort the PDH *q_e_* values.

At first, the adsorption equilibrium was studied in aqueous solution at *I* → 0 mol L^−1^ and *T* = 293.15 K. Then, the thermodynamic study was extended by evaluating the effects of (i) interacting and non-interacting media (NaNO_3_ or NaCl 0.1 mol L^−1^), (ii) organic ligands with different functional groups (MA, Ac, and Cys at *c* = 5 10^−4^ mol L^−1^), and (iii) temperature (284.15 ≤ *T* (K) ≤ 303.15).

The equilibrium pH (pH_f_) of isotherm experiments carried out in water and in solution with backgrounds that do not interact with lead ions (NaNO_3_ 0.1 mol L^−1^, and MA 5 10^−4^ mol L^−1^, the latter, totally protonated at pH = 5) was equal to or, at most, slightly higher than the initial pH (ΔpH up to 0.3 units). When an interacting background was added to the Pb^2+^ solution (NaCl 0.1 mol L^−1^, Cys or Ac 5 10^−4^ mol L^−1^), the pH_f_ increased by almost one unit. The pH_f_ values of suspensions containing only the backgrounds (without Pb^2+^ ions) (see the pH values in parentheses reported in Table 5) were, on average, one unit higher than 5 as a consequence of protonation of some functional groups of PDH. The presence of Pb^2+^ ions hinders the protonation of PDH sites (the pH_f_ is almost the same as that in parentheses) only when the background interacts with the toxic metal ion (NaCl, Ac, and Cys), forming more or less stable species.

In all isotherm experiments, the pH_f_ was lower than that of the formation of low-solubility Pb^2+^ hydrolytic species.

**Figure 10:**
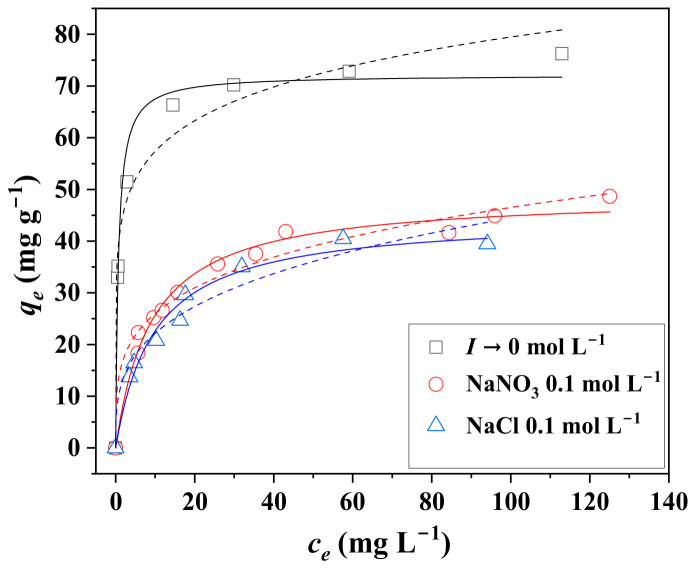
Adsorption isotherms of Langmuir (continuous line), and the Freundlich (dashed line) equations for the adsorption of Pb^2+^ ions by PDH particles in aqueous solution at pH = 5.0, without ionic medium and with the addition of NaNO_3_ or NaCl 0.1 mol L^−1^, and *T* = 293.15K.

The experimental data (*q_e_* vs. *c_e_*) were fitted with the Langmuir and the Freundlich isotherm equations. Results of regression analysis with both isotherm models are depicted in Figure 10 and in Figures S1 and S2 of the Supplementary Materials. The relevant fitting parameters are collected in Table 5 together with the experimental maximum adsorption capacity (*q_m exp_*), the adj. R^2^, and the standard deviations of the fits. Better fitting was achieved by means of the Langmuir model.

The maximum adsorption capacity of PDH at *I* → 0 mol L^−1^ was 72 mg g^−1^. The presence of an ionic medium, regardless of its interaction with lead, reduced the adsorption ability of PDH to the same magnitude (*q_m_* = 49 and 45 mg g^−1^ in NaNO_3_ and in NaCl, respectively) (see Figure 10).

It can be attributed to the shielding effect of the ions deriving from the salts, and to the competition towards PDH binding sites of the Na^+^ ions, whose concentration is at least 2 orders of magnitude larger than that of Pb^2+^ ions.

The formation of Pb–Cl species in NaCl, which slightly reduces the percent of lead positively charged species (% of Pb^2+^ = 50.6, % of PbCl_3_^−^ = 0.8, % of PbCl_2_ = 10.3, % of PbCl^+^ = 38.3 at pH = 5.0, in NaCl 0.1 mol L^−1^ and *T* = 298.15 K), can explain the small reduction in the *q_m_* value in this ionic medium. Both ionic media drastically reduced the affinity of PDH towards the toxic metal ion and, consequently, worsened its adsorption ability at low Pb^2+^ concentration (*K_L_* = 1.4, 0.11, and 0.10 L mg^−1^ at *I* → 0 mol L^−1^, in NaNO_3_ 0.1 mol L^−1^, and NaCl 0.1 mol L^−1^, respectively).

These results are consistent with those reported by Zhao et al. (2022) [68], who observed a similar decrease in the Pb^2+^ adsorption onto synthetic humic acid when NaNO_3_ was added to the toxic metal ion solution. The further reduction in the adsorption capacity of PDH in NaCl is consistent with what was found by Fuentes et al., Lascari et al., and Cataldo et al. for the adsorption of the same toxic metal ion onto microcrystalline cellulose beads, *Opuntia ficus indica* cladodes, and almond shell, respectively [26,27,69].

The organic ligand addition markedly affected the adsorption capability of PDH. The maximum adsorption ability (*q_m_*) trend was Cys > Ac >> H_2_O > MA, whereas the affinity (*K_L_*) trend was H_2_O >> MA >> Ac ≈ Cys.

The amino groups of MA, added to the Pb^2+^ solution at a much lower concentration than the two ionic media previously discussed, are fully protonated at pH = 5 and do not interact with the Pb^2+^ ion [70]. The slight reduction of *q_m_* and the halving of the *K_L_* value can be attributed to the same shielding effect previously discussed for the two ionic media. The significantly lower MA concentration justifies the smaller reduction of *q_m_* and *K_L_* values.

The effect of Ac and Cys on the Pb^2+^ adsorption was completely different. Indeed, the binding groups of both ligands are partially deprotonated at pH = 5.0 and can bind Pb^2+^ ions. The formation of Pb^2+^ species with both organic ligands in the pH range investigated was confirmed by the distribution diagrams built with literature formation constants [71,72] and at the same experimental conditions of adsorption experiments, using PyES v1.1.3 software [73] (see Figure S3 of Supplementary Materials). The Pb^2+^ complexation drastically reduces the concentration of Pb^2+^ aquo ion and, consequently, the affinity of PDH towards the toxic metal ion with *K_L_* values lower than 0.1 L mg^−1^. However, both organic ligands increased the maximum adsorption ability of PDH (*q_m_* = 93 and 101 mg g^−1^ for Ac and Cys, respectively).

The increase of *q_m_* can be tentatively attributed to the adsorption of the organic ligands onto the PDH particles, which creates new adsorption sites available to bind the Pb^2+^ ions.

The comparison between the maximum adsorption capacity (in mmol g^−1^) and the binding groups density of PDH_1_ (likely carboxylic groups) from potentiometric titrations (see Section 3.1) indicates that the stoichiometric ratio between mmol of Pb^2+^ adsorbed and mmol of binding groups per gram of PDH_1_ went from 1.5:1 in NaNO_3_ 0.1 mol L^−1^ to 3.4:1 when Cys 5 ×10^−4^ mol L^−1^ is added to the Pb^2+^ solution. These findings evidenced that the binding groups of PDH_1_ moieties are not the only sites involved in the Pb^2+^ adsorption. This agrees with the interpretation of the FT-IR difference spectrum (see Figure 5) that suggested an involvement of aromatic moieties and of polyphenols of PDH during Pb^2+^ adsorption. The highest stoichiometric ratios found in the presence of Ac and Cys (3.1:1 and 3.4:1, respectively) can be tentatively explained in terms of co-participation of the functional groups of the ligands adsorbed on the surface of PDH [74].

Literature data of Pb^2+^ adsorption onto the two by-products derived from the almond industry, namely shell and hull, are reported in Table S2 of Supplementary Materials, together with the relevant experimental conditions [20,23,26,38,75]. Almond shell (PDS) was the most investigated and showed *q_m_* and *K_L_* values that did not exceed 25.546 mg g^−1^ and 0.34 L mg^−1^, respectively [20,38,75,76].

To the best of our knowledge, only Nasseh et al. studied the Pb^2+^ adsorption onto an almond hull-based adsorbent [23]. However, the authors investigated a composite material formed by hull and Fe_2_O_3_ without evaluating the contribution of the pristine almond hull to the adsorption. Moreover, the authors did not report the percentage of Fe_2_O_3_ in the composite material. The Pb^2+^ adsorption isotherm followed the Langmuir model in agreement with the PDH here investigated. The very low adsorption capacity and affinity (*q_m_* = 5.76 mg g^−1^ and *K_L_* = 0.53 L mg^−1^ at pH = 5 and at *I* → 0 mol L^−1^) can be attributed, to a lesser extent, to the different almond cultivar of the hull (*Prunus amygdalus*—Fascionello) but, mostly, to the addition of Fe_2_O_3_. This indicates the superior Pb^2+^ recovery efficiency of the raw material and the advantage of an adsorbent preparation process that does not require the use of additional reagents.

The results obtained here show that PDH is a better adsorbent of lead ions than PDS, with a markedly higher maximum adsorption ability and affinity (*q_m_* = 72 mg g^−1^ and *K_L_* = 1.4 L mg^−1^ at pH = 5 and at *I* → 0 mol L^−1^). This can be explained in terms of different chemical composition and lignocellulosic structure, with PDH having a higher content of labile polysaccharide fractions and a lower lignin content, as evidenced by the comparison of their TGA curves (see Section 3.1).

### 3.4. Effect of Temperature of Pb^2+^ Adsorption onto PDH

The effect of temperature on Pb^2+^ removal was investigated by performing adsorption isotherms at pH = 5.0 in 0.1 mol L^−1^ NaNO_3_, covering the temperature interval 284.15–303.15 K (see Figure S1 of Supplementary Materials). Examination of the *qm* values derived from Langmuir fitting (Table 6) showed that the adsorption capacity of PDH increased slightly as the temperature increased.

The effect of the temperature on the adsorption of metal ions onto biomasses is known to vary markedly among different systems. In some cases, increasing temperature results in higher adsorption capacities, typically due to the activation or generation of additional adsorption sites. In other systems, increasing temperature causes a reduction in adsorption performance, often attributable to thermally induced degradation of the surface functional groups responsible for metal binding [77,78].

In the present study, the thermal behavior of PDH, as evidenced by its TGA profile (Figure 6), did not show significant changes across the investigated temperature range, indicating the absence of structural degradation. Therefore, the slight enhancement of *q_m_* value at higher temperatures was attributed to an increased availability of accessible superficial binding sites.

**Figure 11:**
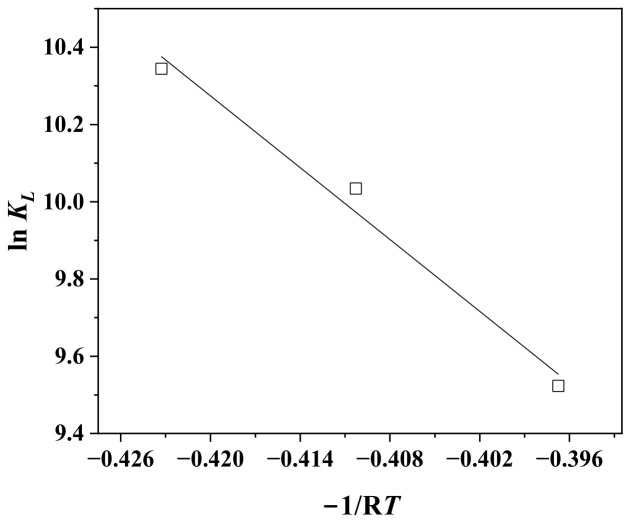
Plot of ln*K_L_* vs. −1/R*T* for the calculation of thermodynamic parameters Δ*H*^0^ and Δ*S*^0^ for the Pb^2+^ ions adsorption onto PDH at pH = 5.0, in NaNO_3_ 0.1 mol L^−1^, in the temperature range 284.15–303.15 K, by using the van’t Hoff equation.

The thermodynamic parameters Δ*G*^0^, Δ*H*^0,^ and Δ*S*^0^ for Pb^2+^ adsorption onto PDH were determined by applying the Gibbs and van’t Hoff equations to the Langmuir constants obtained at each temperature (Table 6). Prior to these calculations, the Langmuir equilibrium constants (*K*_L_), originally expressed in L mg^−1^, were converted to L mol ^−1^ to ensure compatibility with thermodynamic formalism. Moreover, the Langmuir constants were converted into dimensionless constants by multiplying *K_L_* by the standard concentration C° = 1 mol L^−1^. The van’t Hoff plot showed a good linear correlation (Figure 11), suggesting that the enthalpy did not change over the studied temperature range. The resulting Δ*H*^0^ value (−31 kJ mol^−1^) confirmed the exothermic nature of Pb^2+^ adsorption onto PDH. The magnitude of Δ*H*^0^, being well below the commonly accepted |40 kJ mol^−1^| threshold, as well as the Δ*G*^0^ values, suggested a predominant physisorption mechanism [79,80,81].

### 3.5. Breakthrough Adsorption Curves

**Figure 12:**
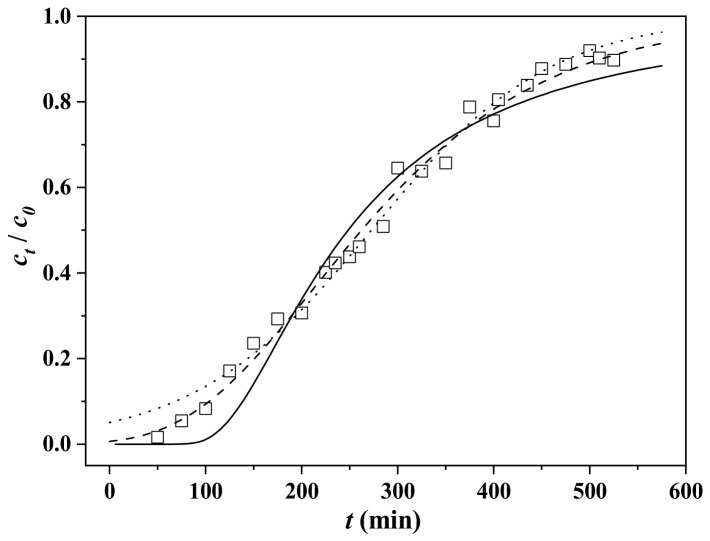
Breakthrough curves of logistic (dot line), Gompertz (dashed line), and Log-Gompertz (continuous line) models for the Pb^2+^ ions adsorption on PDH in aqueous solution at pH = 5.0, *T* = 293.15 K, *c*_0_ = 5 mg L^−1^, and flow rate 3.25 mL min^−1^.

Fixed-bed column experiments were carried out at different experimental conditions (see Section 2). The breakthrough data *c_t_*/*c*_0_ vs. *t* were analyzed by nonlinear regression using the Logistic, the Gompertz, and the Log-Gompertz models. The experimental data together with the fit curves obtained with the three models are reported in Figure 12 and in Figures S4 and S5 of the Supplementary Materials. The parameter values of the models and the breakthrough times at *c_t_*/*c*_0_ = 0.5 (BT_0.5_) are reported in Table S3 of Supplementary Materials.

In all the column experiments, Pb^2+^ ions were initially adsorbed by PDH. As the adsorbent approached saturation, the breakthrough curves asymptotically approached unity. The typical nearly symmetric sigmoidal profile (S-shaped) of the breakthrough curve obtained in water was not observed when the Pb^2+^ solution contained background salts. Indeed, in both the ionic media, the profile was asymmetric or characterized by a gradually increasing curve depending on the electrolyte composition.

The Gompertz model was the best breakthrough model in terms of experimental data fit, except for the data in NaCl 0.1 mol L^−1^, for which the Log-Gompertz model showed the highest adj. R^2^ value (see Table S3).

The empirical nature of the models does not allow using their parameters in the evaluation of breakthrough curves. However, the comparative parameter BT_0.5_, i.e., the breakthrough time at which *c_t_*/*c*_0_ = 0.5, can be calculated.

**Figure 13:**
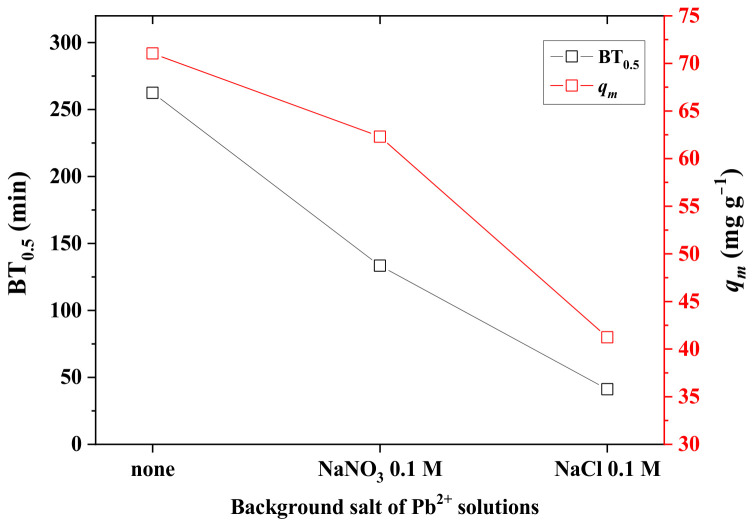
*q_m_* and BT_0.5_ values in pure water, in NaNO_3_ 0.1 mol L^−1^, and in NaCl 0.1 mol L^−1^.

The highest BT_0.5_ value was observed in the absence of an ionic medium. The addition of background salt at a concentration of 0.1 mol L^−1^ reduced the BT_0.5_ value in a similar way and for the same reasons seen for the *q_m_* parameter of the batch adsorption isotherm (see Figure 13), confirming that the adsorption behavior of PDH particles under equilibrium and dynamic, non-equilibrium conditions is comparable.

### 3.6. Recycle and Reuse Experiments

**Figure 14:**
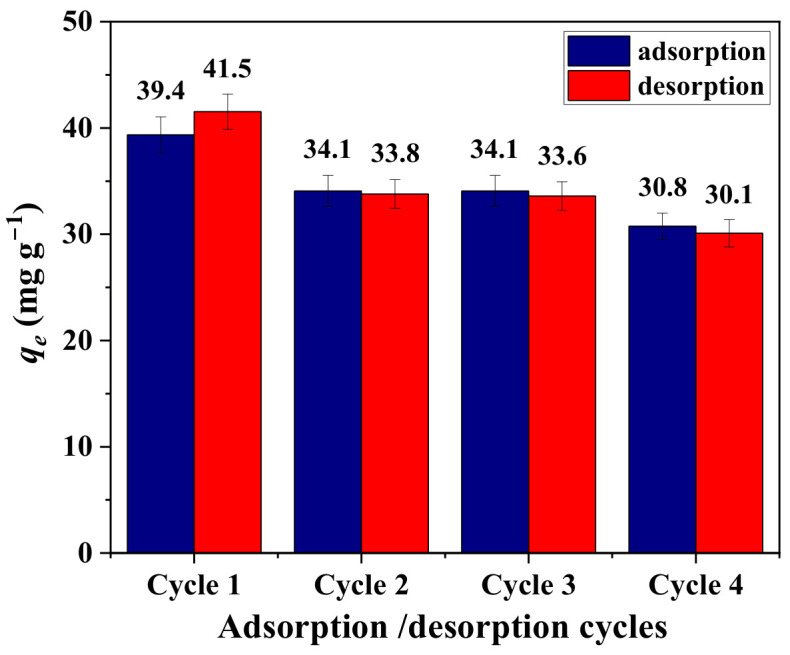
*q_e_* values of adsorption/desorption cycles of Pb^2+^ ions (V = 15 mL, *c*_Pb2+_ = 65 mg L^−1^) on PDH particles (19.9 mg). Experimental conditions: pH = 5.0, *T* = 293.15 K, extractant solution: 15 mL of HCl 0.1 mol L^−1^.

Decontamination process costs can be significantly reduced when the adsorbent material is reusable. For this reason, recycle experiments in a column have been carried out, monitoring the adsorption performance of PDH particles after four adsorption/desorption cycles (see Section 2). The trend of adsorption and desorption *q_e_* values in the four cycles is reported in Figure 14. After the first cycle, PDH underwent a slight decrease in adsorption capacity (~13%) and maintained the same *q_e_* value also in the third cycle. A further slight worsening was registered in the fourth cycle (~9%). The adsorption trend highlighted a good reusability of the adsorbent, whose performance, in terms of reusability, is in line with that of other biomasses [27]. In each cycle, the *q_e_* of adsorption and desorption reached the same values within the experimental uncertainties. The complete Pb^2+^ desorption in each adsorption/desorption cycle is a further indication that Pb^2+^ adsorption can be mainly considered a physisorption process.

### 3.7. Cost-Effectiveness and Environmental Impact of the Adsorbent

Adsorption onto biomass is considered one of the most suitable and eco-friendly procedures for wastewater decontamination. However, the adsorption performance of biomass is often improved through chemical or physical modifications (e.g., pyrolysis, acid or alkaline treatments, magnetization) or multi-step preparation routes [82]. While effective, these treatments can considerably increase production costs, energy demand, and environmental impact [83], making it essential to carefully evaluate each preparation step when developing low-cost adsorbents [84].

A preliminary laboratory-scale cost assessment was performed to estimate the production cost of 1 kg of PDH, encompassing raw material collection, washing, mechanical treatment, drying, and grinding [85]. The total estimated cost was €1.62 kg^−1^ (Table S4, Supplementary Materials), with deionized water (€0.60 kg^−1^) and oven drying (€0.58 kg^−1^) as the principal cost contributors. The adoption of solar drying as an initial dehydration step significantly reduced both energy consumption and associated costs. Electricity costs were calculated at €0.17 kWh^−1^, based on the current average rate for non-domestic supply in Italy [86]. Furthermore, the demonstrated reusability of the adsorbent for at least two adsorption cycles (see Section 3.6) could reduce the overall production cost by approximately 50%, further enhancing the economic feasibility of the material.

Direct comparison of PDH production costs with literature data remains challenging, as reported values are highly dependent on the reagents employed, the processing steps considered, and local electricity prices [87]. Nevertheless, available data provide a useful benchmark. Alsulaili et al. [88] reported laboratory-scale production costs of $0.48–0.75 kg^−1^ for several agricultural waste-derived adsorbents, while more extensively processed materials from similar precursors, such as activated carbons, reached $8.67 kg^−1^. Within this comparative framework, and accounting for its reusability, the estimated production cost of PDH falls within the range reported for biomass-based adsorbents.

A more informative comparison can be drawn with bergamot pomace (BP), a similar lignocellulosic by-product previously investigated by our research group [84] under analogous preparation conditions. The laboratory-scale production cost of BP was estimated at € 14.76 kg^−1^, approximately nine times higher than that of PDH. The main cost drivers for BP were the large volumes of deionized water required for washing and the energy-intensive drying process. In contrast, the PDH protocol was optimized by replacing the initial deionized water washes with tap water, reserving deionized water for the final rinse only, and by introducing a preliminary solar drying step prior to oven drying.

The suitability of PDH is further supported by the origin of the raw material. Indeed, *Prunus dulcis* hull is a by-product of the agri-food industry of the Mediterranean area and is available in abundance. Moreover, the production protocol of PDH does not contain chemical reagents and, in general, hazardous substances, thereby reducing the environmental burden associated with treatment and disposal.

## 4. Conclusions

The *Prunus dulcis* hull furnished by the local agri-food industry has been tested as an adsorbent material for Pb^2+^ ions. The adsorption equilibrium was reached within ~700 min with a well-defined kinetic of adsorption, well defined by the PFO model. The poor linearity of data fit obtained with the Weber–Morris kinetic model does not completely exclude diffusion phenomena, although other interaction processes related to a physisorption mechanism predominate. The affinity and the adsorption capacity of PDH towards Pb^2+^ ions depend on the pH, the ionic medium, and the temperature, as well as on the presence of organic ligands possibly present in the aqueous solution to be treated. The differences in the adsorption behavior of PDH were explained in terms of (i) shielding effect and competition of the ions coming from the background dissociation, (ii) acid-base properties of functional groups of PDH, and (iii) chemical speciation of Pb^2+^ ions. The outcomes of characterization experiments suggest the involvement of aromatic moieties and polyphenols in the biomass interaction with Pb^2+^ ions. The adsorption isotherms were well described by the Langmuir equation. The highest affinity was found at pH = 5.0, *I* → 0 mol L^−1^ and *T* = 284.15 K (*K_L_* = 1.4 L mg^−1^) whilst the maximum adsorption capacity was found in solutions containing Cys (*q_m_* = 101 mg g^−1^). The adsorption process was spontaneous and exothermic. The magnitude of Δ*G*^0^ and Δ*H*^0^ suggested a physisorption mechanism. The adsorption behavior of PDH under equilibrium (batch experiments) and non-equilibrium conditions (column experiments) was comparable. The adsorption capacity of PDH after four adsorption–desorption cycles decreases by only 22%, which denotes excellent reusability. Thanks to the easy preparation, PDH is a cost-effective adsorbent and has no impact on the environment.

## Figures and Tables

**Table 1:** Results obtained from the potentiometric titrations of PDH in NaCl 0.1 mol L^−1^, at *T* = 298.15 K, in the pH range of 2–5, assuming only one monoprotic functional group (PDH_1_).

Mass (g)	log *K*^H^	*c*_PDH1_ ^a^	[H]_T_ ^b^	MD_fit_ ^c^
0.15289		1.04	8.2	
0.20279	3.73 ± 0.02	1.13	7.8	1.6
0.30013		1.63	7.6	

^a^ in mmol L^−1^; ^b^ analytical concentration (mmol L^−1^) of proton obtained by the acid-base titration; ^c^ mean deviation of the fit (in mV).

**Table 2:** Elemental composition of PDH particles as they are and after Pb^2+^ ion adsorption from EDX spectra.

Samples	Elemental Composition (*w*/*w* %) ^a^			
	C	O	Ca	Pb
PDH	68.3 ± 0.7 ^b^	31.3 ± 0.6 ^b^	0.4 ± 0.2 ^b^	-
PDH-Pb^2+^	65.4 ± 1.1 ^b^	29.5 ± 2.3 ^b^	0.8 ± 0.4 ^b^	4.3 ± 0.6 ^b^

^a^ Average of 3 EDX spectra; ^b^ ± std. dev.

**Table 3:** Attribution of ATR-FTIR signals.

Signal (cm^−1^)	Attribution
3335	O-H str.
2920, 2853	Aliphatic C-H str.
1727	C=O str. (-COOH)
1606 (convolution), 1515	Aromatic C=C str. (overlapped with protein Amide-I bands)
1427, 1370, 1317, 1240	C-O str.
1028	C-O-C str. (cellulose, hemicellulose)

**Table 4:** Parameters of PFO and PSO kinetic models for Pb^2+^ adsorption onto PDH particles in aqueous solution containing NaNO_3_ or NaCl 0.1 mol L^−1^, at pH = 5.0, and at *T* = 298.15 K.

Kinetic Model	Medium	*q_e exp_* ^a^	*q_e_* ^a^	*k* ^b^	adj. R^2^	Std. Dev.
PFO	NaNO_3_	30.33	29.8 ± 0.2	(4.69 ± 0.01)×10^−3^	0.9938	0.7645
PSO			36.4 ± 0.3	(1.36 ± 0.01)×10^−4^	0.9950	0.6829
PFO	NaCl	25.9	25.4 ± 0.1	(5.82 ± 0.01)×10^−3^	0.9944	0.6338
PSO			30.0 ± 0.2	(2.23 ± 0.01)×10^−4^	0.9934	0.6889

^a^ mg g^−1^; ^b^ kinetic constant of PFO (min^−1^) and PSO (g mg^−1^ min^−1^) models.

**Table 5:** Freundlich and Langmuir isotherm parameters for the Pb^2+^ ions adsorption on PDH particles in aqueous solution at pH = 5.0, without ionic medium, in NaNO_3_, and in NaCl 0.1 mol L^−1^, in the temperature range 284.15–303.15 K.

					Langmuir Model				Freundlich Model			
Background	*I* ^a^	pH_f_ ^b^	*T*	*q_m exp_* ^c^	*q_m_* ^d^	*K_L_* ^e^	adj. R^2^	Std. Dev.	*K_F_* ^f^	*n*	adj. R^2^	Std. Dev.
none	*I* → 0	5.1 (6.1)	293.15	76	72 ± 2	1.4± 0.2	0.9801	3.7244	41 ± 2	7.1 ± 0.8	0.9737	4.2848
NaNO_3_	0.1	5.3 (5.9)	284.15	40	43 ± 1	0.15 ± 0.02	0.9898	1.2782	17 ± 2	5.0 ± 0.9	0.9681	2.2614
	0.1	5.2	293.15	48.7	49 ± 1	0.11 ± 0.01	0.9819	1.8376	14 ± 1	3.9 ± 0.3	0.9698	2.3730
	0.1	5.2	303.15	49.4	55 ± 1	0.066 ± 0.006	0.9922	1.3235	15 ± 1	3.9 ± 0.4	0.9819	2.0223
NaCl	0.1	5.9 (5.7)	293.15	40.5	45 ± 2	0.10 ± 0.02	0.9753	2.0864	11 ± 1	3.3 ± 0.4	0.9517	2.9168
MA	5 × 10^−4^	4.9 (6.3)	293.15	68	66 ± 2	0.7 ± 0.1	0.9702	3.9445	44.7 ± 0.4	11.6 ± 0.3	0.7304	3.4275
Ac	5 × 10^−4^	5.7 (5.5)	293.15	93	93 ± 2	0.098 ± 0.007	0.9876	2.8974	28 ± 2	4.2 ± 0.4	0.9525	5.6688
Cys	5 × 10^−4^	5.9 (6.1)	293.15	87	101 ± 5	0.037 ± 0.006	0.9752	5.1694	19.2 ± 0.3	3.36 ± 0.03	0.9382	4.7605

^a^ in mol L^−1^; ^b^ mean pH value at adsorption equilibrium; in parentheses the equilibrium pH of the same solutions without Pb^2+^ ions; ^c^ effective maximum adsorption capacity (highest *q_e_* experimental value of the isotherm); ^d^ mg g^−1^; ^e^ L mg^−1^; ^f^ L^1/n^ g^−1^ mg^1−1/n^.

**Table 6:** Thermodynamic parameters for the Pb^2+^ ions adsorption on PDH particles from aqueous solution at pH = 5.0, in NaNO_3_ 0.1 mol L^−1^, in the temperature range 284.15–303.15 K.

*T* (K)	−Δ*G*^0 a^	Δ*H*^0 a^	Δ*S*^0 b^
284.15	24.5 ± 0.5	−31 ± 4	−0.02 ± 0.01
293.15	24.5 ± 0.5		
303.15	23.9 ± 0.5		

^a^ kJ mol^−1^; ^b^ kJ mol^−1^ K^−1^.

## Data Availability

The original contributions presented in this study are included in the article/Supplementary Materials. Further inquiries can be directed to the corresponding author.

## Supplementary Materials

The following supporting information can be downloaded at https://www.mdpi.com/article/10.3390/molecules31132311/s1, Table S1: Stepwise acidic constants of PDH assuming two, three and four sites; Figure S1: Adsorption isotherms of Pb^2+^ onto PDH particles from aqueous solution at pH = 5.0, in NaNO_3_ 0.1 mol L^−1^, in the temperature range 284.15–303.15 K; Figure S2: Adsorption isotherms of Pb^2+^ onto PDH particles from aqueous solution at pH = 5.0 containing MA 0.5 mmol L^−1^ (a), Ac 0.5 mmol L^−1^ (b), and Cys 0.5 mmol L^−1^ (c), at *T* = 293.15 K; Figure S3: Distribution diagrams of Pb^2+^ species vs pH at *I* → 0 mol L^−1^ and at *T* = 298.15 K for Pb–Ac (a) and Pb–Cys (b) systems; Table S2: Literature data of Pb^2+^ ions adsorption onto adsorbents derived from by-products of almond production; Figure S4: Breakthrough curve of Pb^2+^ adsorption onto PDH (90 mg) in NaNO_3_ 0.1 mol L^−1^ at pH = 5.0, *T* = 293.15 K, *c*_0_ = 5 mg L^−1^ and flowrate 3.25 mL min^−1^; Figure S5: Breakthrough curve of Pb^2+^ adsorption onto PDH (90 mg) in NaCl 0.1 mol L^−1^ at pH = 5.0, *T* = 293.15 K, *c*_0_ = 5 mg L^−1^ and flowrate 3.25 mL min^−1^; Table S3: Logistic, Gompertz and Log-Gompertz model parameters and BT_0.5_ values for the Pb^2+^ ions adsorption onto PDH (90 mg) from aqueous solution at pH = 5.0, without ionic medium, in NaNO_3_ 0.1 mol L^−1^ and in NaCl 0.1 mol L^−1^, at *T* = 293.15 K, *c*_0_ = 5 mg L^−1^ and with a flow rate 3.25 mL min^−1^; Table S4: Cost estimation for the lab-scale production of 1 kg of PDH.
