# Supplementary material for: On the Pb2+ Ions Adsorption onto Prunus dulcis Hull
Source: Molecules. 2026 Jul 1;31(13):2311. doi: 10.3390/molecules31132311 (PMC13362795; doi:10.3390/molecules31132311)
Supplement: Supplementary file 1 [file molecules-31-02311-s001.zip › molecules-4334237-supplementary.pdf]

Supplementary Materials

On the Pb^{2+} ions adsorption onto *Prunus dulcis* hull

Davide Lascari [1, †], Salvatore Giovanni Michele Raccuia [2,†], Paolo Lo Meo [3], Nicola Muratore [1], Salvatore Cataldo [1,4], Gabriele Lando [2], Marilena Tolazzi [5], Andrea Melchior [5], Josè Luis Barriada [6], Maria Martinez-Cabanas [6] and Alberto Pettignano [1,4,*]

1 Dipartimento di Fisica e Chimica - Emilio Segrè, Università di Palermo, Viale delle Scienze, I-90128 Palermo, Italy;

2 Dipartimento di Scienze Chimiche, Biologiche, Farmaceutiche ed Ambientali, Università degli Studi di Messina, Viale F. Stagno d'Alcontres 31, I-98166 Messina, Italy;

3 Dipartimento di Scienze e Tecnologie Biologiche, Chimiche e Farmaceutiche, Università di Palermo, Viale delle Scienze, ed. 17, I-90128 Palermo, Italy;

4 NBFC, National Biodiversity Future Center, Palermo, Piazza Marina 61, I-90133 Palermo, Italy

5 Laboratorio di Tecnologie Chimiche, Dipartimento Politecnico di Ingegneria e Architettura, Università di Udine, Udine, I-33100, Italy;

6 Department of Chemistry University of A Coruña, A Coruña, I-15071 Spain

* Correspondence: alberto.pettignano@unipa.it; Tel: +39-091-23897959

† These authors contributed equally to this work.

Supplementary Materials on the acid-base properties of PDH particles

To determine the effective number of proton-active functional groups present on the PDH material, potentiometric titration data were analysed using acid–base models of increasing complexity, including two, three, and four independent monoprotic sites. For each model, the acidity constants (log K^H), the refined concentrations of the active sites (c_{PDHi}), and the mean deviation of the fit were evaluated.

The four-site model yielded the best numerical performance (MD_{fit} =0.9 mV); however, the refined concentration of the most basic site was unrealistically high (up to 74 mmol L^{-1}), and the site densities obtained for different PDH masses were inconsistent. This suggests that such model is unreliable.

The three-site model exhibited similar limitations: the third site (log K^H =12.3) was again characterised by concentrations with no physical meaning (e.g., 146 mmol L^{-1} corresponding to 0.3 g) and a minor fit quality (MD_{fit} = 1.5 mV).

The two-site model further worsened the fit (MD_{fit} =3.5mV) and did not improve the chemical significance of the refined parameters, as the second site remained associated with extremely high logK^H values and unreliable concentrations.

Table S1. Stepwise acidic constants of PDH assuming two, three and four sites. Experimental conditions: NaCl 0.1 mol L^{-1}, T = 298.15 K, pH range: 2 – 11.5.

		PDH_1		PDH_2		PDH_3		PDH_4			
Sites	Mass (g)	log K^H	c_{PDH1}[a]	log K^H	c_{PDH2}[a]	log K^H	c_{PDH3}[a]	log K^H	c_{PDH4}[a]	$[H]_T$[a]	MD_{fit}[c]
	0.15289		0.89		0.15		0.39		67	72	
Four	0.20279	3.38±0.04	1.02	4.83±0.08	0.40	7.76±0.05	0.63	12.23±0.04	74	82	0.9
	0.30013		1.61		0.65		0.69		128	136	
	0.15289		0.90		0.20	-			77	86	
Three	0.20279	3.74±0.01	1.22	7.11±0.04	0.30	-		12.29±0.03	85	94	1.5
	0.30013		1.88		0.40	-			146	154	
	0.15289		1.03	-		-			50	58	
Two	0.20279	3.97±0.02	1.21	-		-		12.08±0.02	58	66	3.5
	0.30013		2.10	-		-			96	104	

[a] in mmol L^{-1}; [b] analytical concentration (mmol L^{-1}) of proton obtained by the acid-base titration; [c] mean deviation of the fit (mV).

Supplementary Materials on the adsorption isotherms

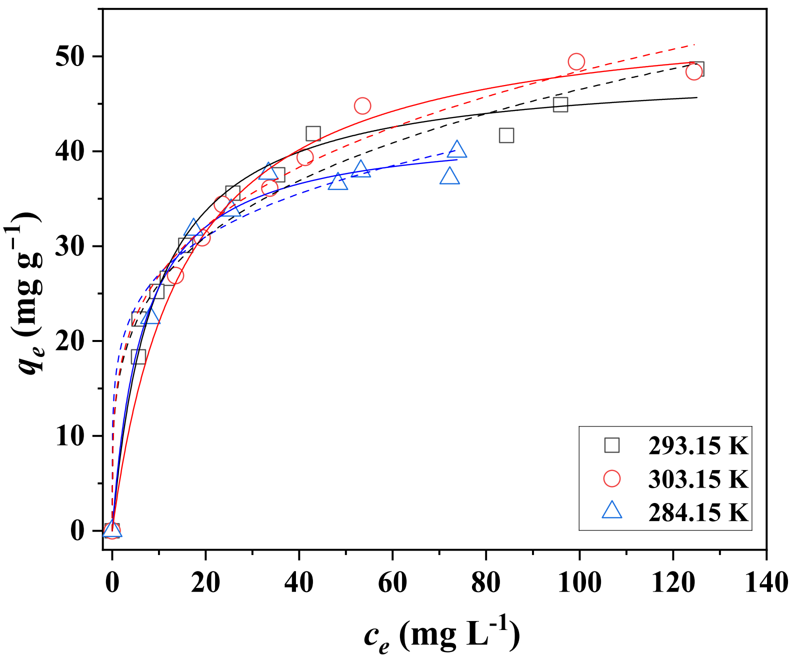

Figure S1 Adsorption isotherms of Pb^{2+} onto PDH particles from aqueous solution at pH = 5.0, in $NaNO_3$ 0.1 mol L^{-1}, in the temperature range 284.15 - 303.15 K. Experimental data were fitted with Freundlich (dashed lines) and Langmuir (continuous lines) isotherm models.

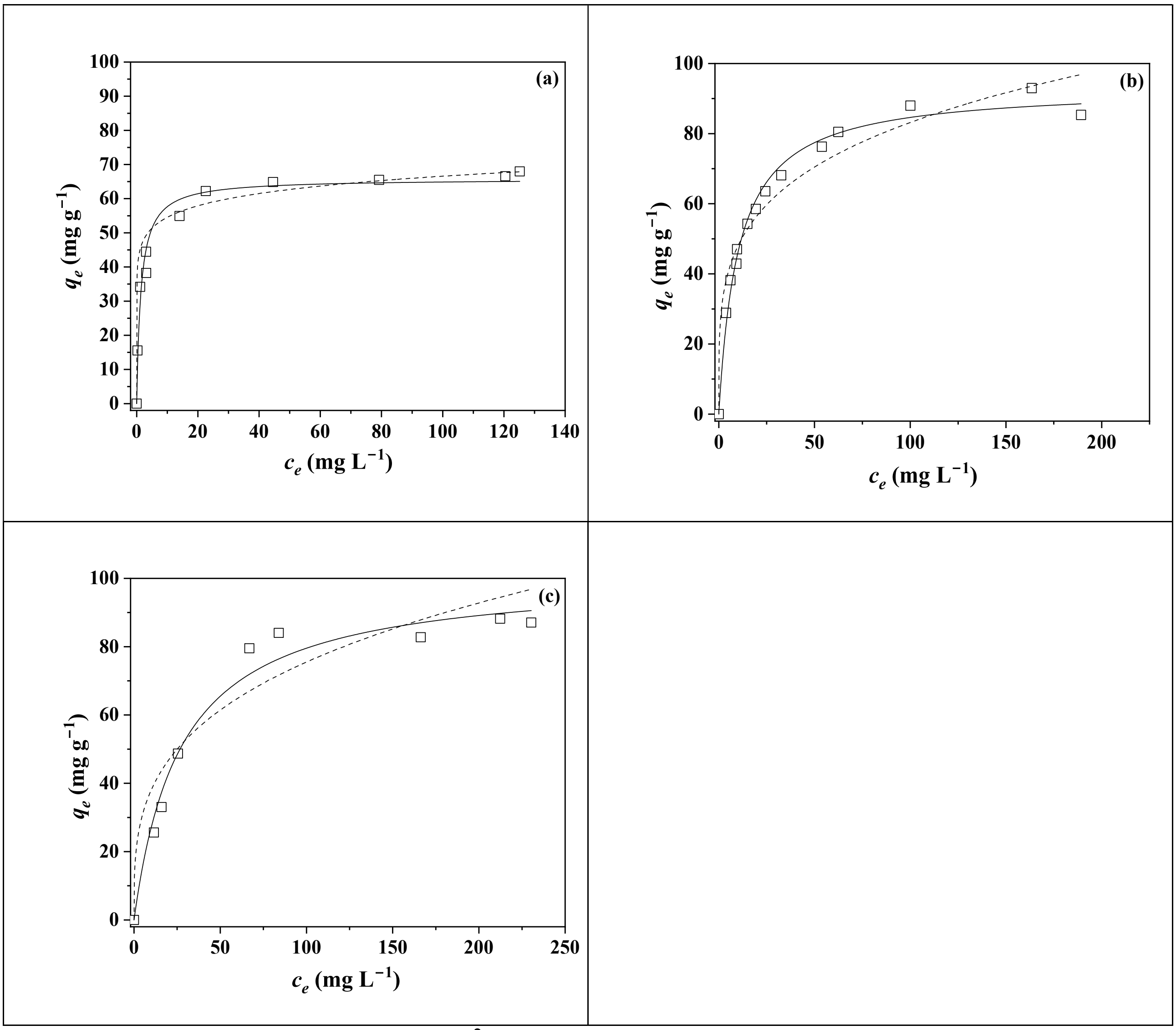

Figure S2 Adsorption isotherms of Pb^{2+} onto PDH particles from aqueous solution at pH = 5.0 containing MA 0.5 mmol L^{-1} (a), Ac 0.5 mmol L^{-1} (b), and Cys 0.5 mmol L^{-1} (c), at T = 293.15 K. Experimental data were fitted with Freundlich (dashed lines) and Langmuir (continuous lines) isotherm models.

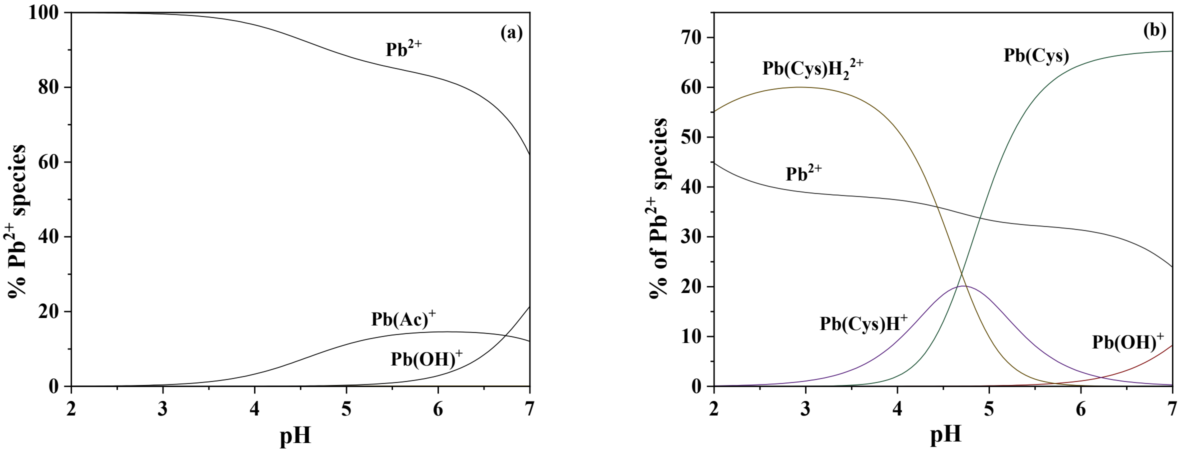

Figure S3. Distribution diagrams of Pb^{2+} species vs pH at $I \rightarrow 0$ mol L^{-1} and at $T = 298.15$ K for Pb – Ac (a) and Pb – Cys (b) systems. Experimental conditions: $c_{Pb2+} = 0.74$ mmol L^{-1}, $C_L = 0.5$ mmol L^{-1}.

Table S2: Literature data of Pb^{2+} ions adsorption onto adsorbents derived from by-products of almond production.

**Adsorbent**	**Dosage** [a]	c_{Pb2+} [b]	**pH$_i$**	T [c]	**Ionic medium**	q_m [d]	K_L [e]	**Ref**
PDS [f)]	10	10 - 200	5.0	298.15	none	5.43	0.011	[75]
PDS	10	20.7 – 200.7	6.0	298.15	KNO_3 [g]	8.08	0.34	[38]
PDS	10	n.d.	5.0	298.15	none	25.546	n.d.	[20]
PDS	5 - 75	30	5.0	298.15	$NaNO_3$ 0.1 mol L^{-1}	5.9	0.05	[26]
PDS	5 - 75	30	5.0	298.15	NaCl 0.1 mol L^{-1}	3.0	0.06	[26]
PDH magnetized with Fe_3O_4	5	10 - 100	9.0	298.15	none	5.76	0.53	[40]
PDH	0.4 - 10	30 - 250	5.0	293.15	none	72	1.4	This work
PDH	0.4 - 10	30 - 250	5.0	293.15	$NaNO_3$ 0.1 mol L^{-1}	49	0.11	This work
PDH	0.4 - 10	30 - 250	5.0	293.15	NaCl 0.1 mol L^{-1}	45	0.10	This work

[a] g L^{-1}; [b] mg L^{-1}; [c] K; [d] mg g^{-1}; [e] L mg^{-1}; [f] Prunus dulcis shell; [g] the background concentration was not specified.

Supplementary Materials on breakthrough curves

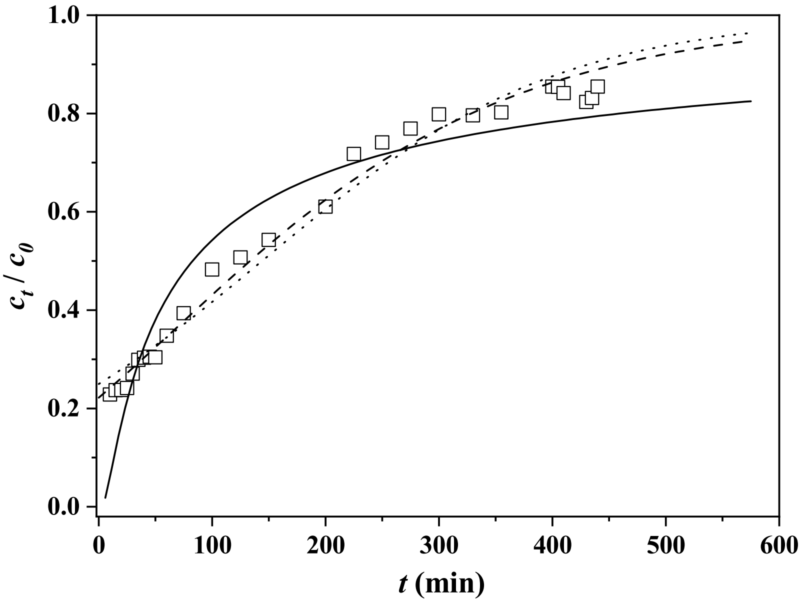

Figure S4 Breakthrough curve of Pb^{2+} adsorption onto PDH (90 mg) in $NaNO_3$ 0.1 mol L^{-1} at pH = 5.0, $T = 293.15$ K, $c_0 = 5$ mg L^{-1} and flowrate 3.25 mL min^{-1}. Experimental data were fitted to Logistic (dot line), Gompertz (dashed line) and Log-Gompertz (continuous line) models.

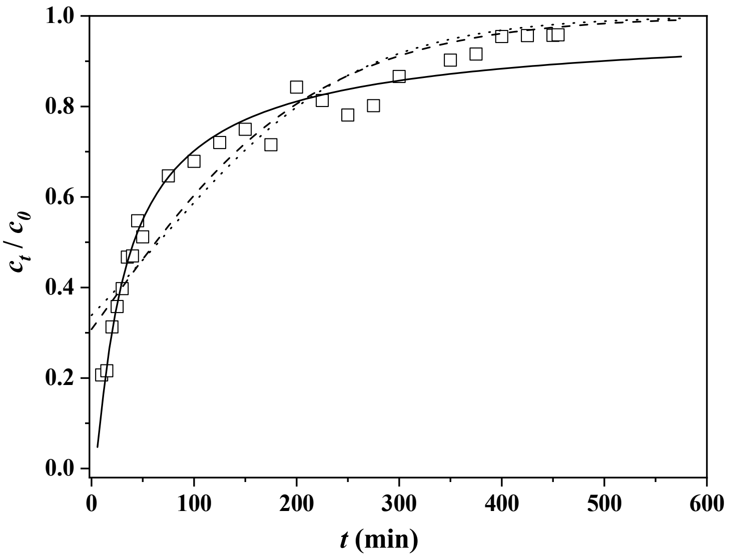

Figure S5. Breakthrough curve of Pb^{2+} adsorption onto PDH (90 mg) in NaCl 0.1 mol L^{-1} at pH = 5.0, T = 293.15 K, c_0 = 5 mg L^{-1} and flowrate 3.25 mL min^{-1}. Experimental data were fitted to Logistic (dot line), Gompertz (dashed line) and Log-Gompertz (continuous line) models.

Table S3. Logistic, Gompertz and Log-Gompertz model parameters and $BT_{0.5}$ values for the Pb^{2+} ions adsorption onto PDH (90 mg) from aqueous solution at pH = 5.0, without ionic medium, in $NaNO_3$ 0.1 mol L^{-1} and in NaCl 0.1 mol L^{-1}, at T = 293.15 K, c_0 = 5 mg L^{-1} and with a flow rate 3.25 mL min^{-1}.

model	parameters	water	$NaNO_3$ 0.1 mol L^{-1}	NaCl 0.1 mol L^{-1}
Logistic	a	2.93 ± 0.13	1.10 ± 0.06	0.67 ± 0.12
	b [a]	0.0107 ± 0.0004	0.0076 ± 0.0003	0.0102 ± 0.0011
	R^2	0.98666	0.97268	0.90333
Gompertz	α_G	1.62 ± 0.07	0.41 ± 0.03	0.16 ± 0.07
	β_G [a]	0.0076 ± 0.0002	0.0058 ± 0.0002	0.0085 ± 0.0008
	R^2	**0.99063** [d]	**0.98579** [d]	0.92259
Log-Gompertz	α_{LG}	11.0 ± 1.5	2.6 ± 0.2	2.4 ± 0.2
	β_{LG} [b]	2.06 ± 0.3	0.66 ± 0.05	0.76 ± 0.04
	R^2	0.96099	0.92311	**0.96817** [d]
	$BT_{0.5}$ [c]	262.37	133.38	41.18

[a] min^{-1}; [b] $(\ln min)^{-1}$; [c] min; [d] best R^2 values.

Supplementary Materials on PDH cost analysis

The steps required to produce 1 kg of PDH adsorbent were as follows:

1) collection of the agricultural waste and its solar drying (free of charge),
2) washing process with tap water (~ 100 L) and deionized water (~10 L),
3) 1,5 hours of mechanical shaking,
4) 48 hours of oven drying at 333.15 K,
5) grinding.

The estimation of the total cost for preparation of PDH required a detailed analysis of any step.
The main resources consumed were electricity and water (in step 2). Therefore, the total cost for PDH production (C_{tot}, in € kg^{-1}) was:

$$C_{tot} = C_{el} + C_{H_2O} \quad (1)$$

where C_{el} and C_{H_2O} are the contribution of electricity and water, respectively, to the total cost. In turn, C_{el} and C_{H_2O} were obtained by multiplying their cost for unit, namely c_{el} (€ kWh^{-1}) and c_{H_2O} (€ L^{-1}) by their estimated consumption, namely E_{tot} (kWh) and V_{H_2O} (L), during the pretreatment process:

$$C_{el} = E_{tot} \cdot c_{el} \quad (2)$$

$$C_{H_2O} = V_{H_2O} \cdot c_{H_2O} \quad (3)$$

The elelctricity consumption was further splitted in three contributions: shaking (E_{shake} , kWh),drying (E_{dry} , kWh) and grinding (E_{grind} , kWh)

$$E_{tot} = E_{shake} + E_{dry} + E_{grind} \quad (4)$$

Electricity consumption for each step was calculated by multiplying the operating power (P, kW) by the working time (t, hour). For the drying stage, the energy demand of the oven was corrected by introducing a duty factor (φ)
The duty factor (φ) represents the fraction of time during which the heating element remains active within a complete cycle to maintain the set temperature. Assuming an initial temperature of 298.15 K and a set temperature of 333.15 K, and considering a maximum operating temperature of 523.15 K for the oven, φ was estimated as:

$$\varphi = \frac{333.15 - 298.15}{523.15 - 298.15} = 0.156 \quad (5)$$

Accordingly, the energy consumption for each step can be expressed by the following equations:

$$E_{\text{shake}} = P_{\text{shake}} \cdot t_{\text{shake}} \quad (6)$$

$$E_{\text{dry}} = P_{\text{oven}} \cdot t_{\text{oven}} \cdot \varphi_{\text{oven}} \quad (7)$$

$$E_{\text{grind}} = P_{\text{grind}} \cdot t_{\text{grind}} \quad (8)$$

The total tap water consumption for the initial washing step was 100 L, while 10 L of deionized water were used for the final washing. The cost of deionized water was estimated based on the laboratory system used to produce it from tap water, taking into account filter replacement and electricity consumption. The resulting unit cost was calculated to be € 0.06 L^{-1}.

Electricity was calculated at € 0.17 kWh^{-1}, based on the current average rate for non-domestic supply in Italy [86]

As a summary, all parameters used for the specific case of the preparation of 1 kg of PDH for the lab scale are reported in Table S4.

Table S4. Cost estimation for the lab-scale production of 1 kg of PDH

Step	Description	Input	Unit	Unit Cost (€)
Collection	Local waste	-	-	-
Solar drying	Free of charge	-	-	-
Initial wash	100 L	0.1 m^3	€ m^{-3} = 1.50	0.15
Final wash	10 L	10 L	€ L^{-1} = 0.06	0.60
Shaking	1.5 h · 0.65 kW	0.975 kWh	€ kWh^{-1} = 0.17	0.17
Drying	48 h · 0.45 kW · 0.156	3.4 kWh	€ kWh^{-1} = 0.17	0.58
Grinding	1 h · 0.7 kW	0.7 kWh	€ kWh^{-1} = 0.17	0.12
				1.62
